# Integrative Multi-Omics Analysis Identifies NUP205 as a Candidate Prognostic Biomarker in Liver Hepatocellular Carcinoma

**DOI:** 10.3390/ijms27062860

**Published:** 2026-03-21

**Authors:** Eun-A Jeong, Jae-Ho Lee, Jongwan Kim

**Affiliations:** 1 Department of Anatomy, Keimyung University School of Medicine, Daegu 42601, Republic of Korea; wjddmsdk2706@naver.com; 2 Department of Anatomy, College of Medicine, Dongguk University, Gyeongju 38066, Republic of Korea

**Keywords:** liver hepatocellular carcinoma (LIHC), prognostic biomarkers, DNA methylation, microRNA (miRNA), bioinformatics analysis

## Abstract

Patients with Liver Hepatocellular carcinoma (LIHC) have a poor prognosis due to late-stage diagnosis and the limited efficacy of drug treatments. Dysregulation of nuclear pore complex (NPC) components, particularly nucleoporins (NUPs), may play a role in tumor progression. However, the specific role of *NUP205* in LIHC has not been comprehensively investigated. We evaluated the expression, prognostic significance, epigenetic regulation, microRNA(miRNA) interactions, drug sensitivity, and biological functions of *NUP205* in LIHC. Comprehensive bioinformatics analyses were performed using publicly available databases and web-based analysis platforms, including The Cancer Genome Atlas (TCGA), UALCAN, and the Kaplan–Meier Plotter (KM Plotter), among others. *In vitro* validation was performed using small interfering RNA (siRNA)-mediated knockdown of *NUP205* in HepG2 cells, followed by quantitative reverse transcription PCR (RT-qPCR), apoptosis assay and wound-healing assay. *NUP205* expression was significantly elevated in patients with LIHC and was associated with advanced clinicopathological features and poor prognosis. Promoter hypomethylation and miRNAs were identified as regulatory mechanisms influencing *NUP205* expression. Increased *NUP205* levels were associated with resistance to multiple chemotherapeutic agents. *NUP205* knockdown significantly reduced messenger RNA (mRNA) expression in HepG2 and PLC/PRF/5 cells, and also reduced the expression of Transmembrane protein 209 (*TMEM209*) in HepG2 cells and improved sensitivity to doxorubicin. *NUP205* expression was consistently associated with adverse clinicopathological features, poor prognosis, and altered drug sensitivity in LIHC. Integrative analyses suggest that *NUP205* dysregulation may be linked to epigenetic and miRNA-associated regulatory mechanisms. These findings support *NUP205* as a candidate prognostic biomarker and a potential regulatory factor in LIHC, warranting further mechanistic and protein-level validation. Further research is necessary to fully elucidate its underlying mechanisms and potential clinical applications.

## 1. Introduction

Liver Hepatocellular carcinoma (LIHC), the most common form of primary liver cancer, represents a significant global health challenge characterized by its increasing incidence and high mortality rates. In 2020, approximately 905,700 new cases of liver cancer were diagnosed worldwide, accompanied by 830,200 deaths, highlighting the persistently poor prognosis [1,2,3]. The principal etiological risk factors for LIHC include chronic infections with hepatitis B and C viruses, excessive alcohol consumption, and metabolic-associated fatty liver diseases, such as non-alcoholic fatty liver disease (NAFLD), which frequently occur in the context of cirrhosis [4,5]. Although advances in vaccination and antiviral therapies have reduced the incidence of virus-related LIHC in certain regions, the overall global incidence continues to increase, partly driven by the NAFLD epidemic and other lifestyle-related factors [5]. Prognosis remains unfavorable, and early-stage LIHC amenable to curative resection or liver transplantation can achieve 5-year survival rates exceeding 70%; the majority of patients present with advanced disease, for which 5-year survival rates fall below 20% [6,7,8]. These clinical challenges underscore the urgent need to identify robust prognostic biomarkers and novel molecular targets for LIHC.

*Nucleoporin 205 (NUP205)* has recently attracted attention as a potential oncogenic factor in several malignancies. *NUP205* is a core structural component of the nuclear pore complex (NPC), which is essential for nucleocytoplasmic transport and maintenance of cellular homeostasis [9,10]. Dysregulation of NPC components has been increasingly linked to tumorigenesis and immune evasion [11]. Among nucleoporins, *NUP205* was prioritized in this study based on preliminary The Cancer Genome Atlas (TCGA) analyses showing consistent upregulation in LIHC and significant associations with adverse clinical outcomes. While several nucleoporins have been implicated in cancer development, the functional and clinical relevance of *NUP205* in LIHC remains insufficiently characterized.

Aberrant overexpression of *NUP205* has been reported in LIHC and other malignancies, including colorectal, bladder, and lung cancers, where it is associated with enhanced tumor progression and poor prognosis [12,13,14]. Functional studies have further suggested that *NUP205* promotes tumor cell proliferation in LIHC models [15]. However, the molecular mechanisms responsible for *NUP205* dysregulation and its contribution to LIHC progression remain largely unclear.

Accumulating evidence indicates that gene dysregulation in LIHC is driven by multiple interacting molecular mechanisms, including epigenetic modifications, genomic alterations, and post-transcriptional regulation. DNA methylation abnormalities, copy-number variations, and microRNA (miRNA)-mediated regulation have each been shown to influence oncogene activation, tumor suppressor silencing, and therapeutic resistance in LIHC [16,17,18,19,20,21]. Importantly, these regulatory layers do not operate independently but instead form interconnected networks that collectively shape tumor behavior. Consequently, integrative multi-omics approaches are essential to fully elucidate the complex regulatory landscape of potential drivers like *NUP205*, capturing complementary alterations that single-omics analyses may miss [22].

The dysregulation of oncogenes in LIHC arises from complex and interdependent molecular mechanisms operating across multiple regulatory layers, including transcriptional, epigenetic, and post-transcriptional processes. Single-omics approaches are often insufficient to fully capture this complexity, as alterations at one molecular level may be compensated or modulated by regulatory events at another. Therefore, integrative multi-omics analyses provide a biologically grounded framework for generating robust hypotheses by revealing coordinated dysregulation across complementary molecular domains [22,23].

In this study, we aimed to elucidate the role of *NUP205* in LIHC through cross-validation using multiple public databases, complemented by validation via in-house experimental assays. Our analyses consistently revealed significant upregulation of *NUP205* expression in LIHC tissues, which was further confirmed at both the messenger RNA (mRNA) and protein levels through laboratory experiments. Elevated *NUP205* expression was strongly associated with poor prognosis, underscoring its potential as a prognostic biomarker for LIHC. Furthermore, we investigated the mechanisms underlying *NUP205* overexpression by examining its relationship with DNA methylation and miRNA regulation. These findings provide an integrative multi-omics-based framework suggesting that *NUP205* dysregulation in LIHC may be influenced by epigenetic and post-transcriptional regulatory mechanisms. This framework offers a rationale for further experimental investigation of *NUP205* as a candidate biomarker and regulatory factor in LIHC rather than definitive mechanistic or therapeutic evidence.

## 2. Results

### 2.1. NUP205 mRNA Expression Is Upregulated in LIHC

**Figure 1:**
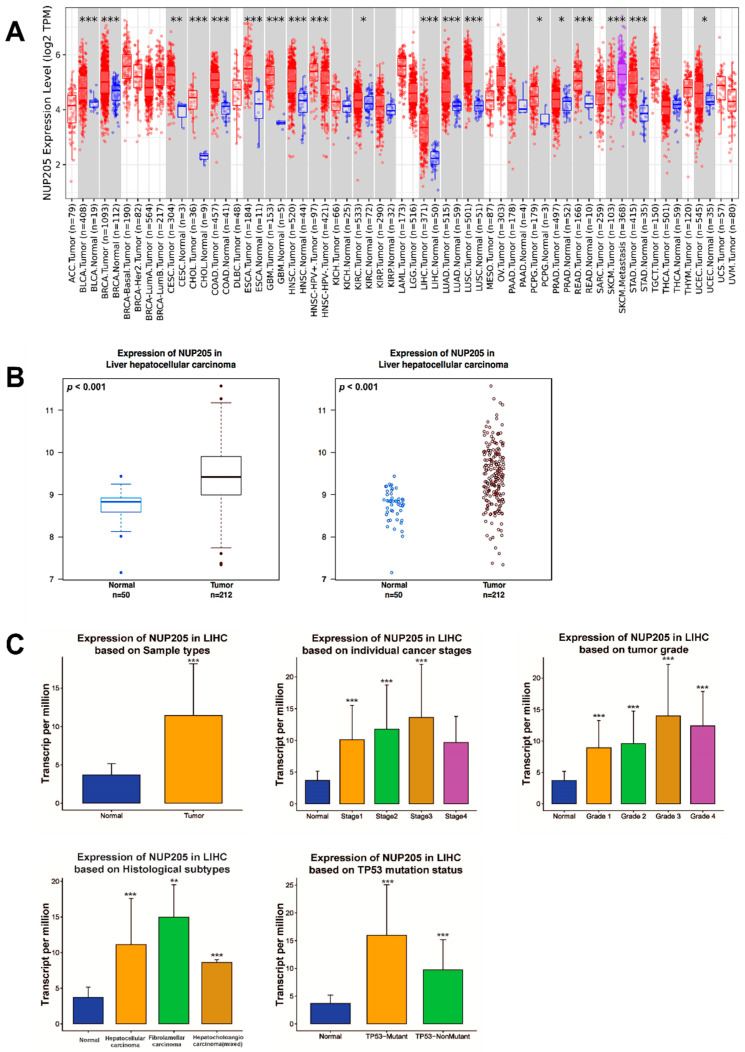
mRNA expression levels of *NUP205* in LIHC. (**A**) Comparison of *NUP205* expression between tumor and normal tissues in various cancer types. (**B**) Comparison of *NUP205* expression between LIHC and normal tissue. (**C**) Comparison of *NUP205* expression between clinicopathologic characteristics and normal conditions. * *p* < 0.05, ** *p* < 0.01, and *** *p* < 0.001.

*NUP205* mRNA expression was markedly elevated in a variety of cancer types relative to normal tissues, including LIHC, bladder urothelial carcinoma, breast invasive carcinoma, cervical squamous cell carcinoma and endocervical adenocarcinoma, cholangiocarcinoma, colon adenocarcinoma, esophageal carcinoma, glioblastoma multiforme, head and neck squamous cell carcinoma (HNSC), Human Papillomavirus (HPV)-positive HNSC, kidney renal clear cell carcinoma, lung adenocarcinoma, lung squamous cell carcinoma, pancreatic adenocarcinoma, pheochromocytoma and paraganglioma, prostate adenocarcinoma, skin cutaneous melanoma–metastasis, stomach adenocarcinoma, and uterine corpus endometrial carcinoma (Figure 1A). To validate these findings in LIHC, *NUP205* expression was further examined using the Wanderer database, which independently confirmed significant upregulation of *NUP205* in LIHC tissues compared to normal tissues (*p* = 0.001) (Figure 1B). *NUP205* expression was assessed across various clinicopathological subgroups within LIHC using the UALCAN database. This analysis demonstrated significant associations between *NUP205* expression and multiple clinical subgroups, including primary tumor, tumor stage (I–III), tumor grade (I–IV), and histological subtype (Figure 1C). The consistent findings across multiple independent datasets underscore the potential utility of *NUP205* as a pan-cancer biomarker and highlight its diagnostic and prognostic relevance, particularly for LIHC.

### 2.2. Upregulated NUP205 Is Associated with Poor Prognosis in LIHC

**Figure 2:**
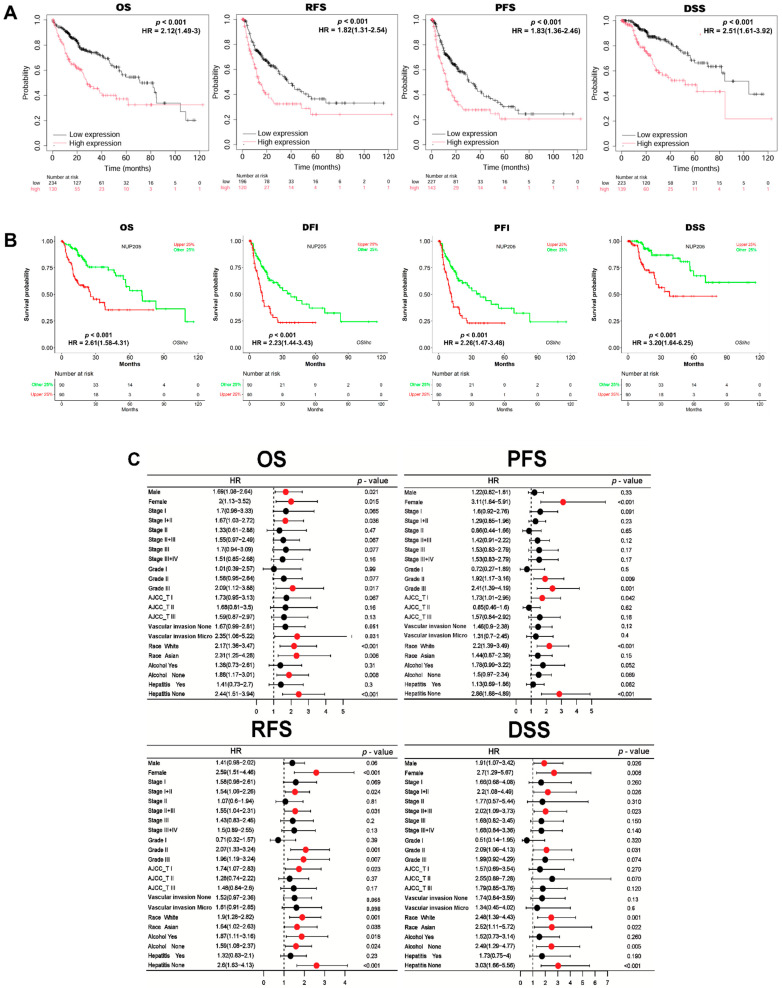
Prognostic significance of *NUP205* expression in LIHC. The prognostic value of *NUP205* expression was analyzed using KM Plotter (**A**) and OSlihc (**B**). (**C**) Clinicopathological features of *NUP205* using KM Plotter. OS: Overall survival, PFS: Progression-free survival, PFI: Progression-free interval, RFS: Relapse-free survival, DFI: Disease-free interval, DSS: Disease-specific survival.

To evaluate the prognostic value of *NUP205* expression in LIHC, we performed survival analyses using the Kaplan–Meier Plotter (KM Plotter) and Online consensus Survival for liver hepatocellular carcinoma (OSlihc) databases. Elevated *NUP205* expression was significantly associated with poor prognosis in LIHC, as demonstrated by worse Overall Survival (OS) (Hazard Ratio [HR]: 2.12, *p* < 0.001), Recurrence-Free Survival (RFS) (HR: 1.82, *p* < 0.001), Progression-Free Survival (PFS) (HR: 1.83, *p* < 0.001), and Disease-Specific Survival (DSS) (HR: 2.51, *p* < 0.001) (Figure 2A). Subsequent analyses confirmed that upregulated *NUP205* expression was significantly associated with poorer prognosis in OS (HR: 2.6081, *p* < 0.001), Disease-Free Interval (DFI) (HR: 2.2252, *p* < 0.001), PFS (HR: 2.26, *p* < 0.001), and DSS (HR: 3.20, *p* < 0.001) (Figure 2B). Further investigation revealed a consistent association between elevated *NUP205* levels and an unfavorable prognosis in patients with LIHC. The relationship between *NUP205* expression and clinicopathological features was also examined, revealing that higher *NUP205* expression was correlated with worse prognosis across various subgroups. Specifically, for OS, increased *NUP205* expression was associated with poorer outcomes in male (HR: 1.69, *p* = 0.021), female (HR: 2.0, *p* = 0.015), stage I+II (HR: 1.67, *p* = 0.036), grade III (HR: 2.09, *p* = 0.017), vascular invasion (micro, HR: 2.35, *p* = 0.031), race (white, HR: 2.17, *p* < 0.001), race (asian, HR: 2.31, *p* = 0.006), alcohol (none, HR: 1.88, *p* = 0.008), and hepatitis (none, HR: 2.44, *p* < 0.001). For PFS, increased *NUP205* expression was associated with poorer outcomes in female (HR: 3.11, *p* < 0.001), grade II (HR: 1.92, *p* = 0.009), grade III (HR: 2.41, *p* = 0.001), American Joint Committee on Cancer (AJCC)_T I (HR: 1.73, *p* = 0.042), race (white, HR: 2.2, *p* < 0.001), and hepatitis (none, HR: 2.86, *p* < 0.001). For RFS, increased NUP205 expression was associated with poorer outcomes in female (HR: 2.59, *p* < 0.001), stage I+II (HR: 1.54, *p* = 0.024), stage II+III (HR: 1.55, *p* = 0.031), grade II (HR: 2.07, *p* = 0.001), grade III (HR: 1.96, *p* = 0.007), AJCC_T I (HR: 1.74, *p* = 0.023), race (white, HR: 1.9, *p* = 0.001), race (asian, HR: 1.64, *p* = 0.038), alcohol (yes, HR: 1.87, *p* = 0.018), alcohol (none, HR: 1.59, *p* = 0.024), and hepatitis (none, HR: 2.6, *p* < 0.001). For DSS, increased *NUP205* expression was associated with poorer outcomes in male (HR: 1.91, *p* = 0.026), female (HR: 2.7, *p* = 0.006), stage I+II (HR: 2.2, *p* = 0.066), stage II+III (HR: 2.02, *p* = 0.023), grade II (HR: 2.09, *p* = 0.031), race (white, HR: 2.48, *p* = 0.001), race (asian, HR: 2.52, *p* = 0.022), alcohol (none, HR: 2.49, *p* = 0.005), and hepatitis (none, HR: 3.03, *p* < 0.001) (Figure 2C). These subgroup analyses should be interpreted as exploratory. Collectively, these results suggest that elevated *NUP205* expression is a robust indicator of poor prognosis in LIHC, underscoring its potential utility as a prognostic biomarker.

### 2.3. NUP205 Is Associated with DNA Methylation in LIHC

**Figure 3:**
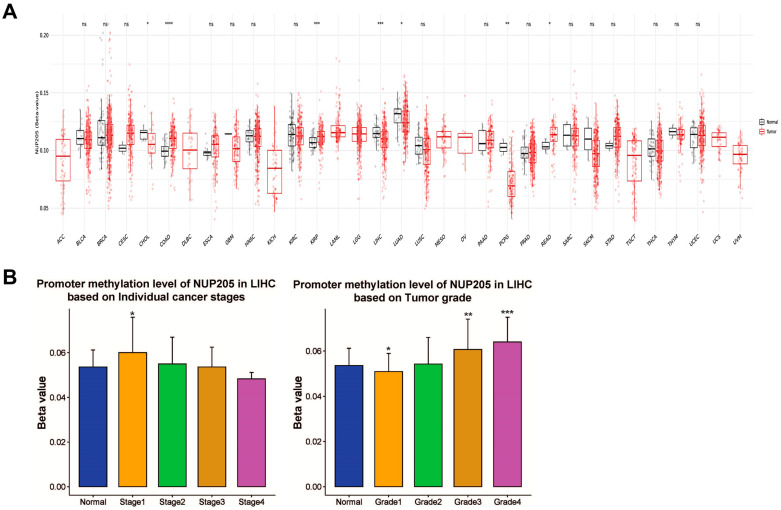
DNA methylation levels of *NUP205* in LIHC. (**A**) Comparison of DNA methylation of *NUP205* between tumors and normal tissues in various cancer types. (**B**) Comparison of *NUP205* DNA methylation between LIHC and normal clinicopathologic characteristics. * *p* < 0.05, ** *p* < 0.01, *** *p* < 0.001 and **** *p* < 0.0001.

**Figure 4:**
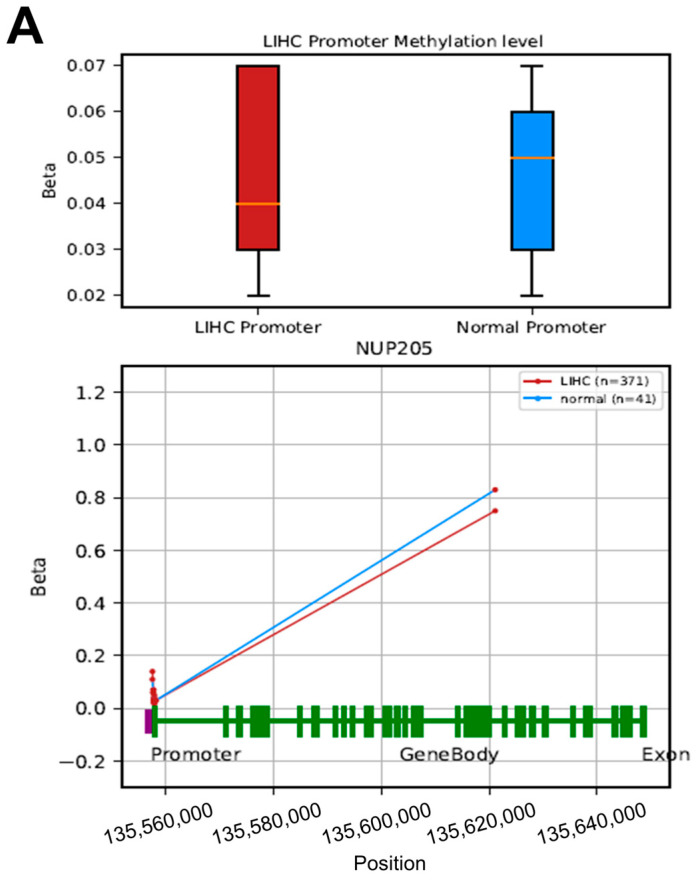

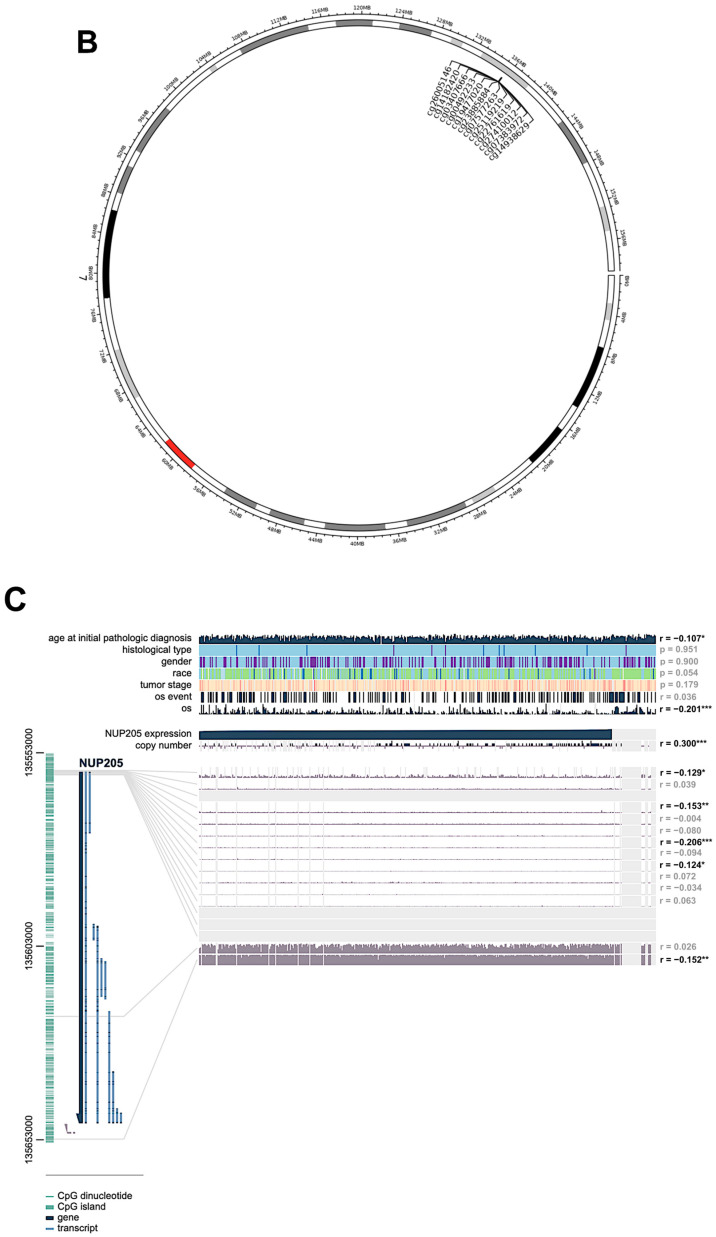
DNA methylation and correlation of *NUP205* in LIHC. (**A**) DNA methylation of *NUP205* between LIHC and normal tissues. (**B**) DNA methylation probes mapped to the *NUP205* in LIHC. (**C**) Correlation of *NUP205*-associated probes in LIHC. * *p* < 0.05, ** *p* < 0.01, and *** *p* < 0.001.

**Figure 5:**
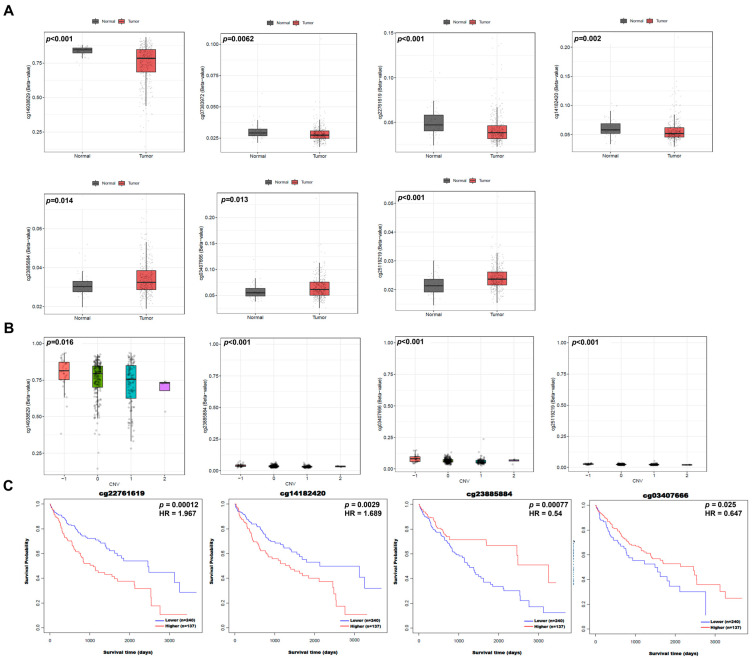
Comparison of *NUP205*-associated DNA methylation probes between LIHC and normal. (**A**) Expression of *NUP205*-associated probes in LIHC. (**B**) Copy-number variations in *NUP205*-associated probes in LIHC. (**C**) Prognostic significance of *NUP205*-associated probes in LIHC.

To elucidate the epigenetic regulation of *NUP205* in LIHC, DNA methylation analyses were conducted using multiple public databases, including UALCAN, OncoDB, SMART, and MEXPRESS. DNA methylation, primarily occurring at cytosine–phosphate–guanine (CpG) sites and catalyzed by DNA methyltransferases (DNMTs), is a key epigenetic mechanism regulating gene expression without altering the DNA sequence. In general, promoter hypermethylation is associated with transcriptional repression, whereas promoter hypomethylation facilitates transcriptional activation. Therefore, the hypomethylation of the *NUP205* promoter observed in this study may contribute to its increased expression in LIHC and may be associated with poor clinical outcomes. In these analyses, promoter regions were defined according to standardized transcription start site (TSS)-centered annotations provided by each TCGA-based platform. These findings indicated significant hypomethylation of the *NUP205* promoter region across various cancer types, including LIHC (Figure 3A). Further investigation revealed that DNA methylation of *NUP205* was significantly associated with clinicopathological characteristics such as pathological stage I and histological grades I, III, and IV in LIHC (Figure 3B). *NUP205* is associated with hypomethylation in LIHC compared to normal tissue and has 12 probes (Figure 4A,B). Correlation analysis demonstrated that methylation at these *NUP205*-associated probes was negatively correlated with DNA methylation levels at several CpG sites, including cg26005146 (R = −0.129, *p* < 0.05), cg03407666 (R = −0.153, *p* < 0.01), cg23885884 (R = −0.206, *p* < 0.001), cg25119219 (R = −0.124, *p* < 0.05), and cg16572020 (R = −0.152, *p* < 0.01) (Figure 4C). Furthermore, probes associated with *NUP205* exhibited hypomethylation in LIHC tissues compared with normal tissues. Several probes, including cg14938629 (*p* < 0.001), cg07383972 (*p* = 0.0062), cg22761619 (*p* < 0.001), and cg19477020 (*p* = 0.002), showed significant hypomethylation in LIHC. In contrast, other *NUP205*-associated probes, such as cg23885884 (*p* = 0.014), cg03407666 (*p* = 0.013), and cg25119219 (*p* < 0.001), exhibited relative hypermethylation in LIHC compared to normal controls (Figure 5A). Analysis of the relationship between these probes and copy-number variations (CNVs) in LIHC revealed that several probes, including cg14938629 (*p* = 0.016), cg23885884 (*p* < 0.001), cg03407666 (*p* < 0.001), and cg25119219 (*p* < 0.001), were significantly associated with CNV alterations (Figure 5B). Moreover, evaluation of the prognostic relevance of *NUP205*-associated probes indicated that cg22761619 (HR: 1.967, *p* < 0.001), cg14182420 (HR: 1.689, *p* = 0.0029), cg23885884 (HR: 0.54, *p* < 0.001), and cg03407666 (HR: 0.647, *p* = 0.0025) were significantly associated with OS, suggesting that hypomethylation at these loci is associated with poorer prognosis (Figure 5C). These findings underscore the potential of *NUP205* as an epigenetic biomarker and suggest its utility in risk stratification, molecular subtyping, and the development of precise epigenetic therapies for LIHC.

### 2.4. NUP205 Is Associated with miRNAs in LIHC

**Figure 6:**
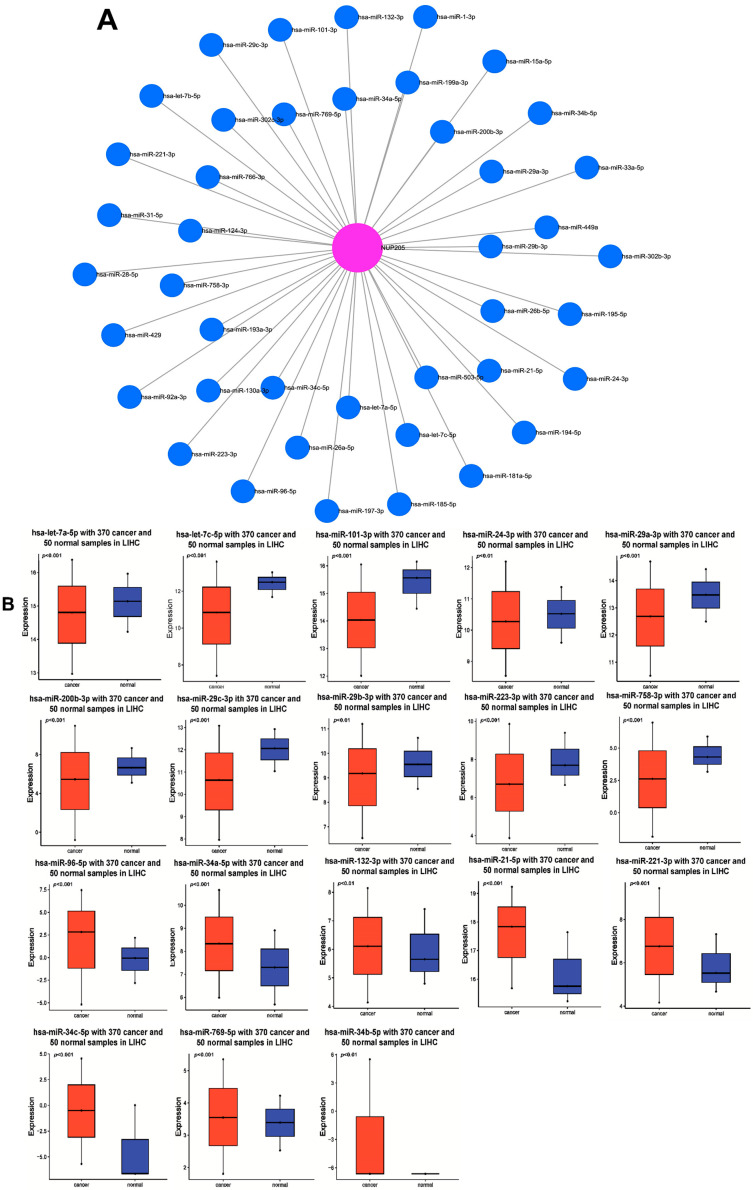

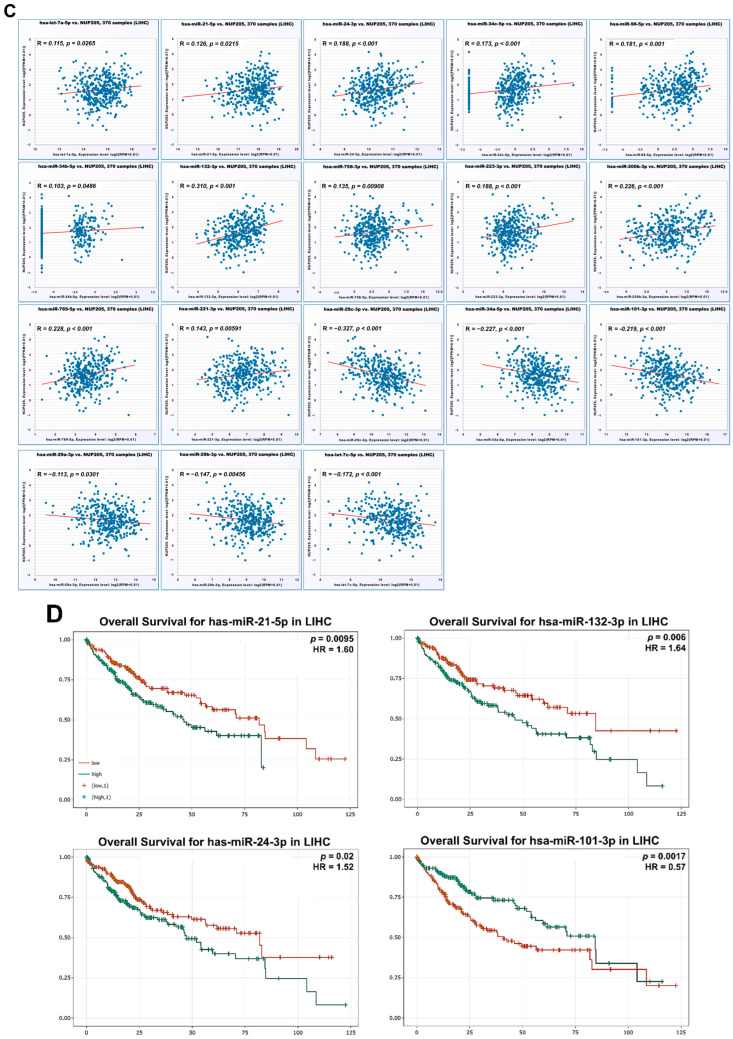
*NUP205*-associated miRNAs in LIHC. (**A**) miRNA network associated with *NUP205*. (**B**) Expression of *NUP205*-associated miRNAs in LIHC. (**C**) Correlation between *NUP205* and *NUP205*-associated miRNAs in LIHC. (**D**) KM plotter of several *NUP205*-associated miRNAs.

To identify miRNAs potentially targeting *NUP205*, predictive interaction analysis was performed using the miRNet database, a comprehensive platform for exploring miRNA–target gene networks. The resulting network comprised 41 miRNAs predicted to interact with *NUP205*, which was positioned as the central node (magenta), while each miRNA was represented as a peripheral node (blue circles) (Figure 6A). To assess the functional impact of *NUP205*-targeting miRNAs on LIHC, candidate miRNAs were analyzed for their expression levels, correlation with *NUP205*, and prognostic significance. To investigate the regulatory relationship between *NUP205* and its target miRNAs in LIHC, we analyzed their differential expression patterns. Several *NUP205*-targeting miRNAs were significantly downregulated in LIHC tumor tissues compared to normal liver tissues, including hsa-let-7a-5p (*p* < 0.001), hsa-let-7c-5p (*p* < 0.001), hsa-miR-101-3p (*p* < 0.001), hsa-miR-24-3p (*p* = 0.0073), hsa-miR-29a-3p (*p* < 0.001), hsa-miR-200b-3p (*p* < 0.001), hsa-miR-29c-3p (*p* < 0.001), hsa-miR-29b-3p (*p* = 0.0085), hsa-miR-223-3p (*p* < 0.001), and hsa-miR-758-3p (*p* < 0.001). Several *NUP205*-associated miRNAs were significantly upregulated in LIHC, including hsa-miR-96-5p (*p* < 0.001), hsa-miR-34a-5p (*p* < 0.001), hsa-miR-132-3p (*p* = 0.0017), hsa-miR-21-5p (*p* < 0.001), hsa-miR-221-3p (*p* < 0.001), hsa-miR-34c-5p (*p* < 0.001), hsa-miR-769-5p (*p* = 0.031), and hsa-miR-34b-5p (*p* = 0.0036) (Figure 6B). Correlation analysis was performed to assess the association between *NUP205* expression and these miRNAs. Positive correlations were observed between *NUP205* and several miRNAs, including hsa-let-7a-5p (R = 0.115, *p* = 0.0265), hsa-miR-21-5p (R = 0.126, *p* = 0.0215), hsa-miR-24-3p (R = 0.188, *p* < 0.001), hsa-miR-34c-5p (R = 0.173, *p* < 0.001), hsa-miR-96-5p (R = 0.181, *p* < 0.001), hsa-miR-34b-5p (R = 0.103, *p* = 0.0486), hsa-miR-132-3p (R = 0.301, *p* < 0.001), hsa-miR-758-3p (R = 0.135, *p* = 0.00908), hsa-miR-223-3p (R = 0.188, *p* < 0.001), hsa-miR-200b-3p (R = 0.226, *p* < 0.001), hsa-miR-769-5p (R = 0.228, *p* < 0.001), and hsa-miR-221-3p (R = 0.143, *p* = 0.00591). In contrast, negative correlations were identified between *NUP205* and hsa-miR-29c-3p (R = −0.327, *p* < 0.001), hsa-miR-34a-5p (R = −0.227, *p* < 0.001), hsa-miR-101-3p (R = −0.219, *p* < 0.001), hsa-miR-29a-3p (R = −0.113, *p* = 0.0301), hsa-miR-29b-3p (R = −0.147, *p* = 0.00456), and hsa-let-7c-5p (R = −0.172, *p* < 0.001) (Figure 6C). To investigate the prognostic value of *NUP205*-targeting miRNAs in LIHC, survival analyses were conducted based on miRNA expression levels. Elevated expression of several miRNAs, including hsa-miR-21-5p (HR: 1.60, *p* = 0.0095), hsa-miR-132-3p (HR: 1.64, *p* = 0.006), and hsa-miR-24-3p (HR: 1.52, *p* = 0.02), was significantly associated with poorer prognosis. Conversely, reduced expression of hsa-miR-101-3p was associated with poor prognosis (HR: 0.57, *p* = 0.0017) (Figure 6D). Collectively, these findings suggest that *NUP205* is intricately regulated by a network of miRNAs in LIHC, some of which exhibit prognostic significance and may represent potential candidates for therapeutic interventions in miRNA-based treatment strategies.

### 2.5. NUP205 Is Associated with Drug Sensitivity in LIHC

**Figure 7:**
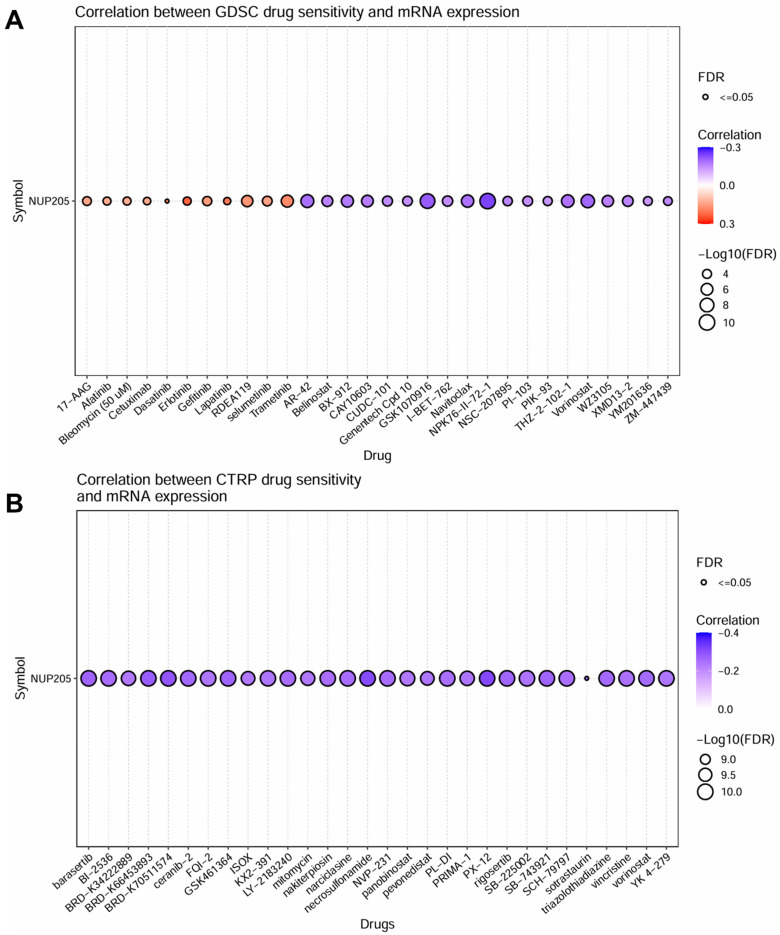
Correlation between *NUP205* expression and drug sensitivity. (**A**) The correlation between *NUP205* and drug sensitivity using GDSC in the GSCA database. (**B**) The correlation between *NUP205* and drug sensitivity using CTRP in the GSCA database. GDSC; Genomics of Drug Sensitivity in Cancer, CTRP; Cancer Therapeutics Response Portal.

To explore the potential therapeutic implications of *NUP205* in LIHC, we investigated the correlation between *NUP205* mRNA expression and drug sensitivity using data from the Genomics of Drug Sensitivity in Cancer (GDSC) and Cancer Therapeutics Response Portal (CTRP) database. The GDSC-based drug sensitivity analysis is shown in Figure 7A. *NUP205* expression was positively correlated with the half-maximal inhibitory concentration (IC_50_) values for agents, including 17-AAG, Afatinib, Bleomycin, Cetuximab, Dasatinib, Erlotinib, Gefitinib, Lapatinib, RDEA119, selumetinib, and Trametinib. This positive correlation indicates that a higher *NUP205* expression may contribute to drug resistance. In contrast, negative correlations were observed between *NUP205* expression and sensitivity to drugs, such as AR-42, Belinostat, BX-912, CAY10603, CUDC-101, Genentech cpd 10, GSK1070916, I-BET-762, Navitoclax, NPK76-II-72-1, NSC-207895, PI-103, PIK-93, THZ-2-102-1, Vorinostat, WZ3105, XMD13-2, YM201636, and ZM-447439. This negative correlation indicates that a higher *NUP205* expression may increase drug sensitivity. These findings suggest that elevated *NUP205* expression may contribute to resistance or sensitivity to distinct classes of targeted therapies, including kinase inhibitors and DNA-damaging agents. The CTRP-based drug sensitivity analysis, depicted in Figure 7B, revealed a significant negative correlation between *NUP205* and sensitivity to a broad range of anticancer compounds, including Barasertib, BI-2536, BRD-K34222889, BRD-K66453893, BRD-K70511574, Ceranib-2, FQI-2, GSK461364, ISOX, KX2-391, LY-2183240, Mitomycin, Nakiterpiosin, Narciclasine, Necrosulfonamide, NVP-231, Panobinostat, Pevonedistat, PL-DI, PRIMA-1, PX-12, Rigosertib, SB-225002, SB-743921, SCH-79797, Sotrastaurin, Triazolothiadiazine, Vincristine, Vorinostat, and YK4-279. This negative correlation indicates that a higher *NUP205* expression may lead to increased drug sensitivity. These findings suggest that elevated *NUP205* expression is associated with increased drug sensitivity in LIHC, particularly to epigenetic modulators, heat shock protein inhibitors, and cyclin-dependent kinase inhibitors. While some of these agents are not currently indicated as primary treatments for LIHC, these findings suggest that *NUP205* may influence drug responsiveness across multiple therapeutic classes, highlighting its potential relevance as a predictive biomarker and a target for further investigation. Collectively, these drug sensitivity analyses indicated that *NUP205* expression may modulate responsiveness to multiple anticancer agents, underscoring its potential role as a predictive biomarker for therapeutic response.

### 2.6. NUP205 Is Associated with Chemicals in LIHC

To investigate the regulatory effects of the chemical compounds on *NUP205* expression, a chemical–gene interaction analysis was conducted using data from the Comparative Toxicogenomics Database (CTD). This analysis identified 66 chemical compounds associated with the upregulation of *NUP205* expression (Table S1), suggesting that they are pharmacological modulators of this gene. Furthermore, the top 21 gene-chemical interaction profiles involving *NUP205* were characterized, revealing a significant overlap with several genes exhibiting similar chemical responsiveness. Notably, nucleoporin 93 (*NUP93*) demonstrated the highest similarity to *NUP205*, with a similarity index of 0.3400 and 51 shared interacting chemicals. Other genes with elevated similarity indices included RNA Binding Motif protein 28 (*RBM28*) (0.3306; 41 chemicals), Testis Expressed 2 (*TEX2*) (0.3206; 42 chemicals), Isoleucyl-tRNA Synthetase 1 (*IARS1*) (0.3118; 53 chemicals), and Timeless Circadian Clock Protein (*TIMELESS*) (0.3099; 53 chemicals). Interestingly, multiple components of the NPC (*NUP93* and *NUP155*) and chromatin-associated regulators Chromatin Assembly Factor 1 Subunit A (*CHAF1A*), Cytoskeleton-Associated Protein 2 Like (*CKAP2L*), and Inner Centromere Protein (*INCENP*) were among the highest-ranked genes, suggesting that *NUP205* may functionally interact with or be co-regulated by similar chemical agents implicated in nuclear transport and chromatin remodeling. Additionally, genes such as Cytoskeleton Associated Protein 4 (*CKAP4*) and FAST Kinase Domains 2 (*FASTKD2*) exhibited substantial chemical interaction overlap (52 and 41 chemicals, respectively), indicating a potentially coordinated transcriptional response to pharmacological perturbations (Table S2). Collectively, these findings delineate a network of chemically coregulated genes associated with *NUP205* and may inform future investigations into shared druggable vulnerabilities within *NUP205*-related molecular pathways.

### 2.7. Gene–Gene Interaction (GGI) Network Analysis of NUP205

Next, a GGI network analysis was performed to elucidate the functional landscape associated with *NUP205*. As shown in Figure S1, the resulting network revealed a highly interconnected regulatory architecture comprising physical interactions, co-expression, co-localization, predicted associations, genetic interactions, shared protein domains, and pathway-level connectivity. *NUP205* occupies a central hub position within this network and interacts directly or indirectly with multiple components of the NPC, including *NUP93*, *NUP98*, *NUP107*, *NUP153*, *NUP155*, and *NUP50*. These associations suggest a coordinated functional role in nucleocytoplasmic transport and the structural organization of the nuclear pore. Furthermore, *NUP205* is connected to key transport-related regulators such as nuclear RNA export factor 1 (*NXF1*), karyopherin subunit beta 1 (*KPNB1*), karyopherin subunit alpha 1 (*KPNA1*), and RAN binding protein 1 (*RANBP1*), indicating its involvement in both mRNA transport and non-coding RNA (ncRNA) export from the nucleus. Functional enrichment analysis revealed that *NUP205* and its interacting partners are significantly associated with mRNA transport, ncRNA export, regulation of gene silencing, and RNA- and miRNA-mediated gene silencing. Collectively, these findings suggest that *NUP205* is embedded within a complex regulatory framework that integrates RNA processing, nuclear transport, and post-transcriptional gene silencing, thereby positioning it as a potential nodal regulator at the interface between transcriptional control and nucleocytoplasmic communication.

### 2.8. Protein–Protein Interaction (PPI) and Functional Enrichment Analysis of NUP205

Next, Protein–Protein Interaction (PPI) network analysis was performed to elucidate the mechanistic role of *NUP205* in biological processes. As shown in Figure S2 (top panel), the STRING-based PPI network identified a cluster of highly interconnected proteins that were functionally associated with *NUP205*. This cluster included *NUP93*, *NUP98*, *NUP107*, *NUP133*, *NUP155*, *NUP160*, *NUP85*, *NUP153*, SEH1-like nucleoporin *(SEH1L)*, and *NUP214*, which are core components of the NPC. To further assess the functional significance of these interacting proteins, Gene Ontology (GO) enrichment analyses were performed across three categories: biological process (BP), cellular components (CC), and molecular functions (MF). In GO-BP, *NUP205*-associated proteins were significantly enriched in RNA- and transport-related processes, including mRNA transport, protein import into the nucleus, nucleocytoplasmic transport, RNA export from the nucleus, and NPC assembly. These findings highlight the involvement of *NUP205* in nucleocytoplasmic trafficking, nuclear organization, and post-transcriptional gene regulation (Figure S2B). GO-CC analysis revealed the predominant localization of these proteins to the nuclear pore, nuclear membrane, nuclear pore inner and outer rings, and central transport channel, consistent with the established structural and transport functions of NPC-associated proteins within the nuclear envelope (Figure S2C). GO-MF analysis revealed the structural constituents of the nuclear pore and the structural molecule activity. Collectively, these results suggest that *NUP205* and its interactors play critical roles in maintaining the integrity and function of the NPC, supporting the hypothesis that *NUP205* functions as a structural scaffold and regulatory mediator of the nucleocytoplasmic transport machinery (Figure S2D).

### 2.9. NUP205 Expression Is Elevated in HepG2 Cells Compared to Other Carcinoma Cell Lines

**Figure 8:**
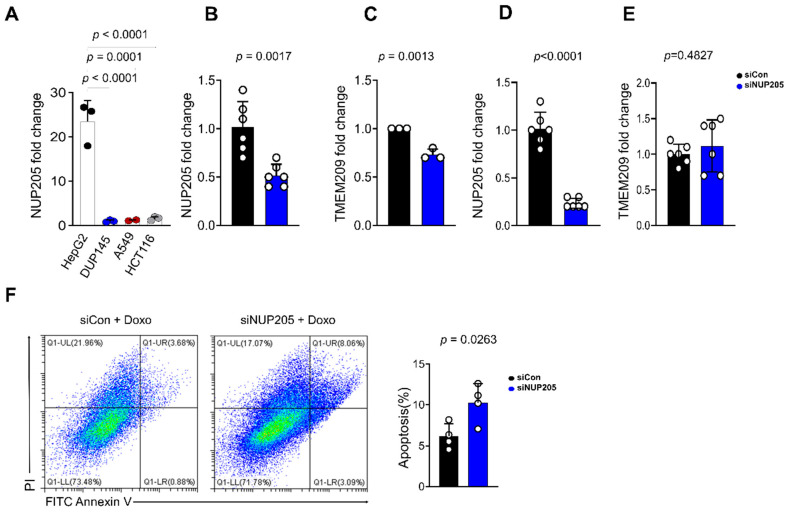
Effects of *NUP205* knockdown in LIHC cells. (**A**) Expression levels of *NUP205* across multiple carcinoma cell lines. (**B**) Knockdown of *NUP205* in HepG2 cells. (**C**) Effect of *NUP205* knockdown on transmembrane protein 209 (*TMEM209*) expression in HepG2 cells. (**D**) Knockdown of *NUP205* in PLC/PRF/5 cells. (**E**) Effect of *NUP205* knockdown on *TMEM209* expression in PLC/PRF/5 cells. (**F**) Apoptotic response to doxorubicin (DOX) when *NUP205* was knocked down in HepG2 cells.

To evaluate the cancer-type specificity of *NUP205* expression, we analyzed its mRNA levels in various carcinoma cell lines, including those derived from prostate, lung, colon, and liver cancers. Notably, *NUP205* expression was significantly higher in HepG2 cells than in other cancer cell lines, such as prostate cancer, lung cancer, and colon cancer (Figure 8A). These findings indicate a potential LIHC-specific role for *NUP205* in tumor biology.

### 2.10. Knockdown Using Small Interfering RNA (siRNA)-NUP205 Reduces Transmembrane Protein 209 (TMEM209) Expression

To elucidate the functional significance of *NUP205* expression in HepG2 cells, we knockdown *NUP205* by transfection with small interfering RNA (siRNA)-*NUP205* and then quantified the transcript level using quantitative reverse transcription PCR (RT-qPCR). Transfection with *NUP205*-specific siRNA resulted in a significant reduction in *NUP205* mRNA expression relative to the control group in HepG2 cells (*p* < 0.01; Figure 8B). Reduces transmembrane protein 209 (*TMEM209*) promotes proliferation, migration, invasion, and epithelial–mesenchymal transition in LIHC cell lines *in vitro*. *TMEM209* enhances proliferation and metastasis of LIHC cells via a karyopherin-β1 (*KPNB1*)-dependent mechanism. Accordingly, the *TMEM209*/*KPNB1*/Wnt/β-catenin signaling axis has been identified as a critical pathway in LIHC progression [24]. Furthermore, *TMEM209* overexpression and *TMEM209*–*NUP205* interaction between *NUP205* have been implicated as key drivers of lung cancer proliferation [12]. Notably, our data showed that *NUP205* knockdown significantly downregulated *TMEM209* expression (*p* = 0.0013; Figure 8C). Additionally, transfection with *NUP205*-specific siRNA resulted in a significant reduction in *NUP205* mRNA expression compared to the control group in PLC/PRF/5 cells (*p* < 0.0001; Figure 8D). However, *NUP205* knockdown does not downregulate *TMEM209* expression (*p* = 0.4827; Figure 8E). These results suggest that *NUP205* may contribute to LIHC progression, at least in part, by modulating *TMEM209* expression.

### 2.11. NUP205 Knockdown Enhances Doxorubicin (DOX)-Induced Apoptosis in HepG2 Cells

**Figure 9:**
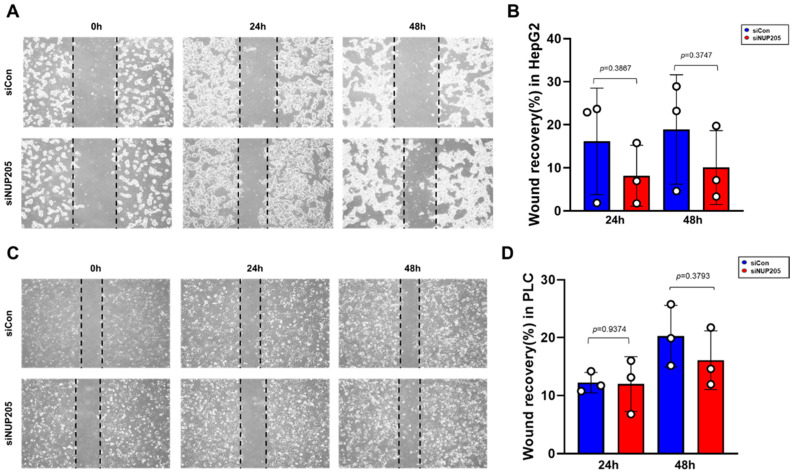
Cell migration in cell scratch wound healing assay. (**A**) Representative images of the wound healing assay performed in HepG2 cells. (**B**) Quantification of wound recovery rates in control and si*NUP205*-treated HepG2 cells. (**C**) Representative images of the wound healing assay performed in PLC/PRF/5 cells. (**D**) Quantification of wound recovery rates in control and si*NUP205*-treated PLC/PRF/5 cells.

To investigate the effect of *NUP205* expression on chemosensitivity, doxorubicin (DOX), a widely used chemotherapeutic agent for various cancers [25,26], was used. Our study demonstrated that the downregulation of *NUP205* in HepG2 cells significantly increased apoptosis (Figure 8F). These findings suggest that *NUP205* plays a role in the drug response mechanisms of LIHC and that its inhibition enhances the apoptotic effect of DOX. Therefore, targeting *NUP205* may represent a promising therapeutic strategy to improve the efficacy of chemotherapy in patients with LIHC. In addition, cell recovery was examined using a wound healing assay. A slight reduction in cell migration was observed in si*NUP205*-treated cells compared with the control group. However, this difference did not reach statistical significance (Figure 9A–D).

## 3. Discussion

In this study, we comprehensively characterized the oncogenic role of *NUP205* in LIHC by integrating multi-omics analyses to elucidate its prognostic significance, epigenetic regulation, miRNA networks, drug sensitivity profiles, and functional interactions. Our results revealed that *NUP205* was significantly upregulated in LIHC, correlated with advanced clinicopathological features, and predicted poor prognosis. Furthermore, mechanistic investigations indicated that the upregulation of *NUP205* is driven by epigenetic alterations and miRNA interactions, which also influence drug sensitivity in LIHC. Moreover, functional validation using *in vitro* assays demonstrated that *NUP205* knockdown was associated with reduced *TMEM209* mRNA expression in HepG2 cells. While this observation suggests a potential regulatory relationship, direct protein-level interactions or causal mechanisms were not assessed in this study.

Based on integrative multi-omics analyses complemented by limited *in vitro* functional assays, we propose a conceptual regulatory framework in which *NUP205* overexpression in LIHC is associated with epigenetic alterations and dysregulated miRNA networks. Rather than establishing a definitive mechanistic model, this framework highlights coordinated molecular associations that may contribute to tumor progression and altered drug responsiveness, which require further validation at the protein and functional levels.

*NUP205* is a key component of the NPC and plays a vital role in nucleocytoplasmic transport and the maintenance of nuclear architecture. Increasing evidence has linked the abnormal overexpression of NPC components to tumorigenesis and immune evasion in various cancer types [11]. Previous studies have identified *NUP205* as an oncogenic factor in several cancers, including colorectal, lung, and nasopharyngeal cancers, where its upregulation is associated with increased proliferation and poor clinical outcomes [12,13,14]. In LIHC, earlier research based on limited datasets suggested a prognostic role for *NUP205* [15,27]. Our study extends these findings by providing a comprehensive multi-omics validation using multiple public databases and functional experiments. The results of this study suggest epigenetic and post-transcriptional mechanisms underlying *NUP205* dysregulation in LIHC, including the identification of DNA methylation patterns and miRNA regulators with prognostic relevance.

Integrative DNA methylation analysis revealed that the upregulation of *NUP205* in LIHC was significantly associated with promoter hypomethylation. This is consistent with previous studies that connected the aberrant epigenetic activation of critical genes to LIHC progression [28,29]. Notably, specific CpG site probes showed inverse correlations with *NUP205* expression and were independently associated with poor prognosis, indicating their potential as differentially methylated markers (DMMs) for risk stratification. These results support previous reports that DNA hypomethylation, especially in cirrhotic or hepatitis B virus (HBV)-infected liver tissues, promotes oncogenic activation and hepatocarcinogenesis [30,31]. Importantly, the biological relevance of *NUP205* upregulation inferred from epigenetic analyses is further supported by our functional experiments, in which *NUP205* knockdown suppressed *TMEM209* expression and enhanced DOX-induced apoptosis in LIHC cell models. While causality between DNA methylation and these downstream phenotypes was not directly tested, these findings collectively suggest that epigenetically associated *NUP205* overexpression contributes to tumor progression and therapeutic response in LIHC.

In addition to epigenetic dysregulation, we identified a network of miRNAs that may regulate *NUP205* expression. Several miRNAs were significantly downregulated in LIHC, whereas oncomiRs, such as hsa-miR-21-5p, hsa-miR-24-3p, and hsa-miR-132-3p, were overexpressed and exhibited a positive correlation with *NUP205* expression. Notably, elevated expression of hsa-miR-21-5p, hsa-miR-24-3p, and hsa-miR-132-3p was associated with poor patient survival, underscoring their potential as prognostic biomarkers and therapeutic targets. Although miRNA–target interactions were initially identified using miRNet, key miRNAs were further supported by cross-validation with The Encyclopedia of RNA Interactomes (ENCORI), which incorporates cross-linking immunoprecipitation sequencing (CLIP-seq)-supported evidence and TCGA-based expression and survival analyses, thereby reducing potential noise from prediction-only approaches. The relatively modest correlation coefficients observed between *NUP205* and several miRNAs are consistent with the multi-layered nature of gene regulation in bulk tumor tissues, where miRNA-mediated effects coexist with epigenetic, transcriptional, and genomic regulatory mechanisms [21,22,32].

Drug therapy remains a significant challenge in enhancing survival outcomes in patients with advanced LIHC [33,34]. Our pharmacogenomic analysis reveals that the upregulation of *NUP205* is associated with responsiveness to various targeted therapies and cytotoxic agents, including kinase inhibitors and epigenetic modulators. DOX, an anthracycline antibiotic derived from *Streptomyces* species, is widely used as an effective chemotherapeutic agent against a range of malignancies, including carcinomas, leukemia, solid tumors, soft-tissue sarcomas, and breast cancer [25,26]. Our findings demonstrate that *NUP205* knockdown sensitizes cells to DOX-induced apoptosis, suggesting a contributory role in drug therapy. However, the clinical application of DOX is limited because of its dose-dependent and irreversible cardiotoxicity [35,36]. Moreover, the precise mechanisms underlying the therapeutic effects of DOX remain a subject of ongoing debate.

GGI and PPI analyses suggested potential associations between *NUP205* and other NPC components involved in nucleocytoplasmic transport. These interaction networks were derived from *in silico* prediction-based databases and should therefore be interpreted as hypothesis-generating rather than as evidence of direct PPIs or regulatory activity in LIHC. Enrichment analyses indicated that genes associated with *NUP205* were enriched in pathways related to mRNA transport, nuclear export, and miRNA-mediated gene silencing. These findings reflect pathway-level associations based on transcriptomic data and do not establish direct mechanistic roles for *NUP205* in these processes.

Despite the advantages of integrating transcriptomic, epigenetic, and functional validation data, this study has several limitations. First, its retrospective design and dependence on publicly available datasets may have introduced a cohort-specific bias. Second, although *in vitro* experiments provided functional evidence, further *in vivo* validation is necessary to confirm the role of *NUP205* in tumor growth and response to drug therapy. In addition, because HepG2 cells are derived from hepatoblastoma rather than hepatocellular carcinoma, findings obtained from this cell line should be interpreted as mechanistic support for the multi-omics analyses rather than definitive evidence of LIHC-specific behavior. Accordingly, the HepG2 cell line was not employed as a definitive disease model of LIHC but rather as an experimental system to explore general mechanisms of gene activity regulation and nucleocytoplasmic transport associated with *NUP205* dysregulation. This clarification is important to avoid overinterpretation of the *in vitro* findings as LIHC-specific phenotypes.

In this context, publicly available functional dependency data from the Achilles/DepMap project provide additional insight. To our knowledge, *NUP205* does not emerge as a strong dependency gene in HepG2 or PLC/PRF/5 cell lines, suggesting that its role may not be universally essential for cell viability. Publicly available dependency datasets indicate that *NUP205* does not represent a strong universal survival dependency in commonly used LIHC cell lines. This finding underscores the context-dependent nature of NUP205 function and supports the interpretation that the observed phenotypes in this study reflect regulatory or modulatory effects rather than essential viability dependency.

Finally, the precise molecular mechanisms by which *NUP205* regulates *TMEM209* or modulates apoptotic pathways remain incompletely understood and warrant further investigation. These limitations reflect the broader analytical scope of the present study. Importantly, the regulatory relationships and biological interpretations proposed in this study are derived from integrative transcriptomic, epigenetic, and *in silico* analyses, complemented by *in vitro* functional assays. Together, the findings provide a framework for hypothesis generation and support future experimental studies aimed at further clarifying these regulatory links.

## 4. Materials and Methods

### 4.1. Data Sources

We conducted a retrospective *in silico* analysis of *NUP205* expression in patients with LIHC. Publicly available multi-omics datasets and online analytical tools were used to investigate gene expression, epigenetic regulation, prognostic value, and interaction networks. The principal data source was TCGA LIHC cohort, comprising approximately 371 tumor samples and 50 adjacent normal liver tissue samples (RNA-seq expression data) [37].

### 4.2. Gene Expression Analysis

To evaluate the mRNA expression levels of *NUP205* in LIHC, we used multiple publicly available bioinformatics platforms, including TIMER2.0, Wanderer, and UALCAN. TIMER2.0 (http://timer.cistrome.org/; accessed on 7 April 2025) is a comprehensive tool for the systematic analysis of immune infiltrates, gene expression, and survival outcomes, incorporating clinical data across various cancer types [38,39]. Wanderer (http://maplab.cat/wanderer; accessed on 7 April 2025) is a user-friendly, gene-centric web application designed to access and visualize TCGA DNA methylation data along with the corresponding mRNA expression across multiple cancer types [40]. UALCAN (http://ualcan.path.uab.edu; accessed on 8 April 2025) was used to compare mRNA expression levels between tumor and normal tissues and to evaluate expression patterns across clinicopathological subgroups, including tumor stage, grade, and *TP53* mutation status [38].

### 4.3. Survival Analysis

To evaluate the prognostic significance of *NUP205* in LIHC, comprehensive survival analyses were performed using multiple publicly accessible bioinformatics platforms, including the KM plotter (http://kmplot.com/analysis/; accessed on 10 April 2025) [38,39] and OSlihc (https://bioinfo.henu.edu.cn/LIHC/LIHCList.jsp; accessed on 12 April 2025) [39]. Both platforms integrate datasets from TCGA, Genotype-Tissue Expression (GTEx), and the Gene Expression Omnibus (GEO). The survival endpoints assessed using the KM plotter included OS, RFS, DSS, and PFS. OSlihc evaluated OS, DFS, RFS, and DSS.

### 4.4. DNA Methylation Analysis

To examine the DNA methylation status of *NUP205* and its prognostic significance in LIHC, several publicly accessible bioinformatics platforms were utilized. The UALCAN platform (methylation module) was used to evaluate the promoter methylation *β*-values of *NUP205* in tumor versus normal tissues, as well as across clinical subgroups. SMART (http://www.bioinfo-zs.com/smartapp/; accessed on 18 April 2025) was used to investigate the inverse correlation between gene expression and DNA methylation across pan-cancer cohorts, facilitating the visualization of expression-methylation relationships by integrating data from TCGA Illumina Human Methylation 450K arrays (Illumina, Inc., San Diego, CA, USA) and RNA-sequencing profiles [41]. OncoDB (https://oncodb.org/; accessed on 22 April 2025) served as an initial screening tool for promoter methylation status in LIHC, enabling the comparison of average β-values between tumors and matched normal tissues [42]. MEXPRESS (https://mexpress.be/; accessed on 28 April 2025) was used to map the methylation levels of CpG sites across the *NUP205* gene locus, aligning each probe with TSS and exon/intron structures, allowing direct comparison between tumor and normal samples while integrating relevant clinical metadata [43]. Finally, MethSurv (https://biit.cs.ut.ee/methsurv; accessed on 29 April 2025) was used to perform univariate Cox regression analyses on individual CpG sites to evaluate their prognostic significance, with KM survival curves generated for each site [44]. DNA methylation data were derived from TCGA Illumina Human Methylation 450K arrays and were obtained in the form of preprocessed β-values as provided by each platform. CpG probes with missing β-values were excluded according to platform-specific pre-processing pipelines, and only probes annotated to the *NUP205* gene region were retained for downstream analyses. In total, 12 *NUP205*-associated CpG probes were included in the final methylation and survival analyses.

### 4.5. Non-Coding RNA Regulatory Network

To investigate the post-transcriptional regulation of *NUP205*, we examined miRNA interactions using miRNet 2.0 and ENCORI (starBase; https://rnasysu.com/encori/index.php; accessed on 7 June 2025). miRNet 2.0 (https://www.mirnet.ca; accessed on 5 June 2025) is a web-based platform designed for constructing and visualizing miRNA-target interaction networks [45]. (Encyclopedia of RNA Interactomes, also known as starBase v3.0) (https://starbase.sysu.edu.cn; accessed on 9 June 2025) provides TCGA expression data for mRNAs and miRNAs, facilitating correlation analyses to identify miRNAs that are inversely correlated with specific mRNAs across cancer samples. Additionally, ENCORI offers survival analyses for these miRNAs in LIHC. For each miRNA potentially targeting *NUP205*, we assessed its prognostic significance using ENCORI’s Kaplan–Meier plots comparing the high and low expression groups [46].

### 4.6. Drug Sensitivity Analysis

The Gene Set Cancer Analysis (GSCA) platform (https://guolab.wchscu.cn/GSCA/#/; accessed on 11 June 2025) integrates TCGA datasets encompassing 33 cancer types and including data on mRNA expression, DNA methylation, somatic mutations, immune infiltration, and drug sensitivity derived from the CTRP and the GDSC [47]. The CTD (http://ctdbase.org; accessed on 12 June 2025) is a manually curated resource that associates genes with chemicals, diseases, and phenotypes, based on evidence extracted from the peer-reviewed literature [48].

### 4.7. Gene Network and Functional Association (GeneMANIA)

To further investigate the genes associated with *NUP205*, we utilized GeneMANIA (http://genemania.org; accessed on 14 June 2025) with both the Cytoscape plugin and web interface. GeneMANIA identifies genes that are functionally similar or related to a query gene by analyzing extensive functional association data, including co-expression, co-localization, protein and genetic interactions, pathways, and domain similarities [49]. It visualizes gene–gene functional interaction networks, integrating data on co-expression, physical interactions, co-localization, and pathway associations.

### 4.8. PPI Network (STRING)

We constructed a PPI network for *NUP205* to identify potential functional associations with other proteins. The STRING database (https://string-db.org; accessed on 16 June 2025) was used to construct a PPI network for *NUP205*. Interacting proteins and enriched GO and Kyoto Encyclopedia of Genes and Genomes (KEGG) terms were retrieved using the default medium confidence threshold [50]. The combined confidence score integrates experimentally validated, curated, and predicted interactions, and only first-shell interactors of *NUP205* were included for downstream functional enrichment analyses.

### 4.9. Cell Culture and siRNA Transfection

HepG2 cells were cultured at 37 °C in a humidified atmosphere containing 5% CO_2_ in Dulbecco’s Modified Eagle’s Medium (DMEM; Thermo Fisher Scientific, Waltham, MA, USA; Cat. No. 11965092) supplemented with 10% fetal bovine serum (FBS; Thermo Fisher Scientific, Cat. No. 16000044) and 1× penicillin–streptomycin (Welgene, Gyeongsan, Republic of Korea; Cat. No. LS20202). PLC/PRF/5 cells were maintained at identical temperature and CO_2_ conditions in RPMI-1640 medium (Welgene, Gyeongsan, Republic of Korea; Cat. No. LM01103) supplemented with the same concentrations of FBS and the antibiotic mixture. Gene silencing was performed using the Lipofectamine RNAiMAX (Thermo Fisher Scientific, Waltham, MA, USA; Cat. No. 13778150) with *NUP205*-specific siRNA or a scrambled negative control (Ambion, Thermo Fisher Scientific, Waltham, MA, USA; siRNA IDs s23176 and s23177) following the manufacturer’s protocols. Forty-eight hours post-transfection, total RNA was extracted using TRIzol reagent (Thermo Fisher Scientific, Waltham, MA, USA; Cat. No. 15596018), and RNA concentration and purity were assessed using a NanoDrop ND-1000 spectrophotometer (Thermo Fisher Scientific, Waltham, MA, USA). The primer sequences used for RT-qPCR analyses are summarized in Table 1.

### 4.10. Quantitative Real-Time PCR (qRT-PCR)

Complementary DNA (cDNA) was synthesized from total RNA using the ReverTra Ace qPCR RT Master Mix (Toyobo, Osaka, Japan; Cat. No. QPK-201) according to the manufacturer’s protocol. The transcript levels of *NUP205*, *TMEM209*, and the reference gene, *GAPDH*, were quantified using the CFX Connect Real-Time PCR Detection System (Bio-Rad, Hercules, CA, USA). The thermal cycling conditions consisted of an initial denaturation at 95 °C for 10 min, followed by 40 cycles of 95 °C for 15 s and 72 °C for 30 s.

### 4.11. Annexin V Staining Assay

HepG2 cells were knocked down with siRNA-*NUP205* and then treated with 2 μM DOX. After 24 h, both the culture supernatant and adherent cells were harvested using a cell scraper and resuspended in phosphate-buffered saline (PBS). The collected cells were then centrifuged at 1500× *g* for 5 min, and the resulting pellet was resuspended in 1× binding buffer (BD Biosciences, Franklin Lakes, NJ, USA; Cat. No. 5166121E). Subsequently, the cells were incubated with FITC-conjugated Annexin V and propidium iodide (PI) (BD Biosciences; Cat. No. 556547) at room temperature in the dark. Apoptotic cells were analyzed using a CytoFLEX S flow cytometer (Beckman Coulter, Brea, CA, USA).

### 4.12. Wound Healing Assay

HepG2 and PLC/PRF/5 cells were seeded in 6-well plates and cultured to 70–80% confluence. Cells were transfected with control siRNA or target-specific siRNA *NUP205* using Lipofectamine 2000. After 6 h, the medium was replaced, and the cells were cultured at 37 °C. A scratch was created in the center of each well, washed with PBS, and incubated in appropriate media (R10 for PLC/PRF/5 and D10 for HepG2). Cell recovery was assessed at 0, 24, 48 h. Cell migration was quantified using the iSolution Lite ×64 software (IMT i-Solution Inc., Vancouver, BC, Canada).

### 4.13. Statistical Analysis

All statistical analyses were performed using integrated tools available on publicly accessible bioinformatics platforms, including TIMER2.0, UALCAN, OSlihc, Kaplan–Meier Plotter, SMART, GSCA, MethSurv, MEXPRESS, ENCORI, miRNet, CTD, GeneMANIA, and STRING. For analyses of gene expression and DNA methylation, either Student’s *t*-test or Wilcoxon rank-sum test was used, according to the data distribution characteristics and platform-specific defaults. Relationships between continuous variables, such as gene expression and DNA methylation levels or gene–miRNA expression pairs, were evaluated using Pearson correlation coefficients. Survival analyses were performed using the log-rank test, and univariate Cox proportional hazards regression models with HRs and 95% confidence intervals (CIs) were used to evaluate prognostic significance. To account for multiple hypothesis testing, Benjamini–Hochberg false discovery rate (FDR) corrections were applied where applicable. A significance threshold of *p* < 0.05 or FDR < 0.05 was applied unless otherwise specified.

To minimize potential batch effects, all transcriptomic, methylation, and miRNA analyses were conducted within the same platform-specific datasets (e.g., TCGA-LIHC) rather than through direct cross-platform data merging. Public databases were used primarily for cross-validation of trends and associations rather than for pooled quantitative analyses. As all datasets were obtained from platforms with standardized normalization pipelines, no additional batch-effect correction was applied.

Network-based functional associations were visualized using Cytoscape version 3.10.1. Experimental data were analyzed using GraphPad Prism software version 3.0 (GraphPad Software, San Diego, CA, USA). Data are presented as the mean ± standard error of the mean (SEM) from at least three independent experiments. Differences between the experimental groups were assessed using two-way analysis of variance (ANOVA), followed by Tukey’s post hoc multiple comparison test to adjust for multiple testing. Statistical significance was set at *p* < 0.05.

## 5. Conclusions

In this study, we identified overexpression of *NUP205* as an important oncogenic driver characterized by adverse prognostic impact and chemoresistance association in LIHC. Integrative analyses demonstrate that *NUP205* expression is associated with promoter hypomethylation and miRNA suppression. Furthermore, functional assays confirmed its role in regulating tumor cell survival and drug response. These findings suggest that *NUP205* may serve as a valuable prognostic biomarker and therapeutic target. Future *in vivo* validation and mechanistic investigations are needed to further elucidate its role in LIHC progression and treatment resistance.

## Figures and Tables

**Table 1:** Primer sequences used for RT-qPCR in this study.

Gene	Primer (5′ to 3′)
*NUP205*	Forward: GATTTTAGAAGTGGGCTGGCT
	Reverse: CGTCTGACAAGAGCCTGTATGA
*TMEM209*	Forward: GCAGACTCACTAAAGTATCCCCA
	Reverse: CTCCATGGTGCTTTTAATGAAG
*GAPDH*	Forward: GAAAGGTGAAGGTCGGAGTC
	Reverse: GTTGAGGTCAATGAAGGGGTC

## Data Availability

The original contributions presented in this study are included in the article/Supplementary Material. Further inquiries can be directed to the corresponding authors.

## Supplementary Materials

The following supporting information can be downloaded at: https://www.mdpi.com/article/10.3390/ijms27062860/s1.

## Abbreviations

The following abbreviations are used in this manuscript:

LIHC	Liver hepatocellular carcinoma
NAFLD	Non-alcoholic fatty liver disease
NUP205	Nucleoporin 205
NPC	Nuclear pore complex
HBV	Hepatitis B virus
DMMs	DNA methylation markers
CNVs	Copy-number variations
miRNAs	MicroRNAs
oncomiRs	Oncogenic microRNAs
mRNAs	Messenger RNAs
DOX	Doxorubicin
TMEM209	Transmembrane Protein 209
TCGA	The Cancer Genome Atlas
KM Plotter	Kaplan–Meier Plotter
siRNA	small interfering RNA
RT-qPCR	quantitative reverse transcription PCR
mRNA	messenger RNA
HNSC	head and neck squamous cell carcinoma
HPV	Human Papillomavirus
OSlihc	Online consensus Survival for liver hepatocellular carcinoma
HR	Hazard Ratio
RFS	Recurrence-Free Survival
PFS	Progression-Free Survival
DSS	Disease-Specific Survival
DFI	Disease-Free Interval
AJCC	American Joint Committee on Cancer
CpG	cytosine–phosphate–guanine
DNMTs	DNA methyltransferases
TSS	transcription start site
GDSC	Genomics of Drug Sensitivity in Cancer
CTRP	Cancer Therapeutics Response Portal
CTD	Comparative Toxicogenomics Database
PPI	Protein–Protein Interaction
GO	Gene Ontology
BP	biological process
CC	cellular components
MF	molecular functions
