# Supplementary material for: Integrative Multi-Omics Analysis Identifies NUP205 as a Candidate Prognostic Biomarker in Liver Hepatocellular Carcinoma
Source: Int J Mol Sci. 2026 Mar 21;27(6):2860. doi: 10.3390/ijms27062860 (PMC13026649; doi:10.3390/ijms27062860)
Supplement: Supplementary file 1 [file ijms-27-02860-s001.zip › ijms-4180824-supplementary.pdf]

Table S1. Interactions between NUP205 expression and chemicals in LIHC using the Comparative Toxicogenomics Database (CTD). Interaction actions indicate qualitative annotations (e.g., increased expression) curated in the CTD; quantitative fold-change values are not provided for chemical–gene interactions in this database.

Chemical Name	Chemical ID	Interaction Actions
1,2-Dimethylhydrazine	D019813	Increases expression
1,4-bis(2-(3,5-dichloropyridyloxy) benzene	C028474	Increases expression
1H,1H,10H,10H-perfluorodecane-1,10-diol	C546883	Increases expression
2,2',3',4,4',5-hexachlorobiphenyl	C029790	Increases expression
2,4,4'-trichlorobiphenyl	C081766	Increases expression
2,4,5,2',4',5'-hexachlorobiphenyl	C014024	Increases expression
2,4,5,2',5'-pentachlorobiphenyl	C009828	Increases expression
2,5,2',5'-tetrachlorobiphenyl	C009407	Increases expression
2-palmitoylglycerol	C114956	Increases expression
4-hydroxyphenyl 4-isopropoxyphenylsulfone	C000613560	Increases expression
7,8-Dihydro-7,8-dihydroxybenzo(a)pyrene 9,10-oxide	D015123	Increases expression
Acetamide	C030686	Increases expression
Acetaminophen	D000082	Increases expression
Aflatoxin B1	D016604	Increases expression
Alpha-Chlorohydrin	D000517	Increases expression
Amitrole	D000640	Increases expression
Antigens, Polyomavirus Transforming	D000952	Increases expression
Aroclors	D001140	Increases expression
Arsenic	D001151	Increases expression
Benzene	D001554	Increases expression
Benzo(a)pyrene	D001564	Increases expression
bisphenol A	C006780	Increases expression
bisphenol B	C492482	Increases expression
bisphenol F	C000611646	Increases expression
Caffeine	D002110	Increases expression
Carbon Tetrachloride	D002251	Increases expression
CGP 52608	C092451	Increases expression
Chlordan	D002706	Increases expression
Copper Sulfate	D019327	Increases expression
Coumestrol	D003375	Increases expression
deoxynivalenol	C007262	Increases expression
Dextran Sulfate	D016264	Increases expression
Erianin	C477638	Increases expression
Flutamide	D005485	Increases expression
furan	C039281	Increases expression
Gentamicins	D005839	Increases expression
hexabromocyclododecane	C089796	Increases expression
Hexachlorocyclohexane	D001556	Increases expression
Hydrocarbons, Chlorinated	D006843	Increases expression
lipopolysaccharide, E coli O55-B5	C482199	Increases expression
methidathion	C005828	Increases expression
Methimazole	D008713	Increases expression
Nanotubes, Carbon	D037742	Increases expression
Okadaic Acid	D019319	Increases expression
PCB 180	C410127	Increases expression
Pentabromodiphenyl ether	C086401	Increases expression
Phenobarbital	D010634	Increases expression
Plant Extracts	D010936	Increases expression
Potassium Dichromate	D011192	Increases expression
propiconazole	C045950	Increases expression
Propylthiouracil	D011441	Increases expression
Resveratrol	D000077185	Increases expression

Rotenone	D012402	Increases expression
Selenium	D012643	Increases expression
Sodium arsenite	C017947	Increases expression
Sodium bichromate	C016104	Increases expression
Sulfadimethoxine	D013412	Increases expression
Thioacetamide	D013853	Increases expression
Toxaphene	D014112	Increases expression
Trimellitic anhydride	C015559	Increases expression
Triphenyl phosphate	C005445	Increases expression
Vinclozolin	C025643	Increases expression

Table S2. Interactions between NUP205-related genes and chemicals in LIHC using. the CTD.

Gene	Similarity Index	Common Interaction Chemicals
NUP93	0.34	51
RBM28	0.3306	41
TEX2	0.3206	42
IARS1	0.3118	53
TIMELESS	0.3099	53
EPS8L2	0.3063	49
FASTKD2	0.306	41
KNTC1	0.3054	51
CHAF1A	0.3049	50
U2SURP	0.3043	42
FXYD1	0.3041	45
RRP1B	0.3034	44
CKAP2L	0.3	48
TBCK	0.2992	38
NUP155	0.2973	44
INCENP	0.2965	51
CKAP4	0.2938	52
HEATR1	0.2938	47
CENPN	0.2937	42
DOCK7	0.2937	42

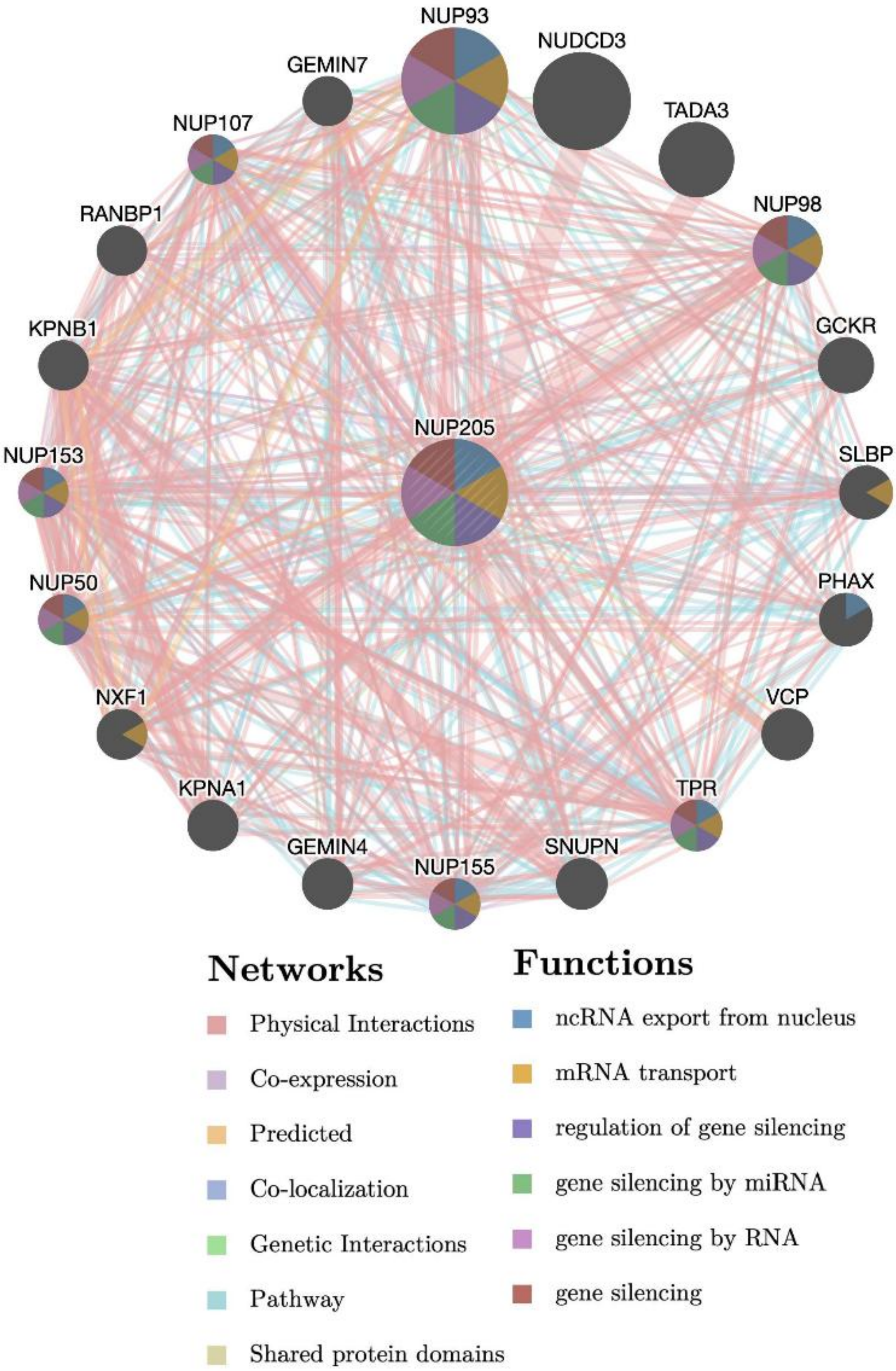

Figure S1. Gene–gene interaction (GGI) network analysis of NUP205.

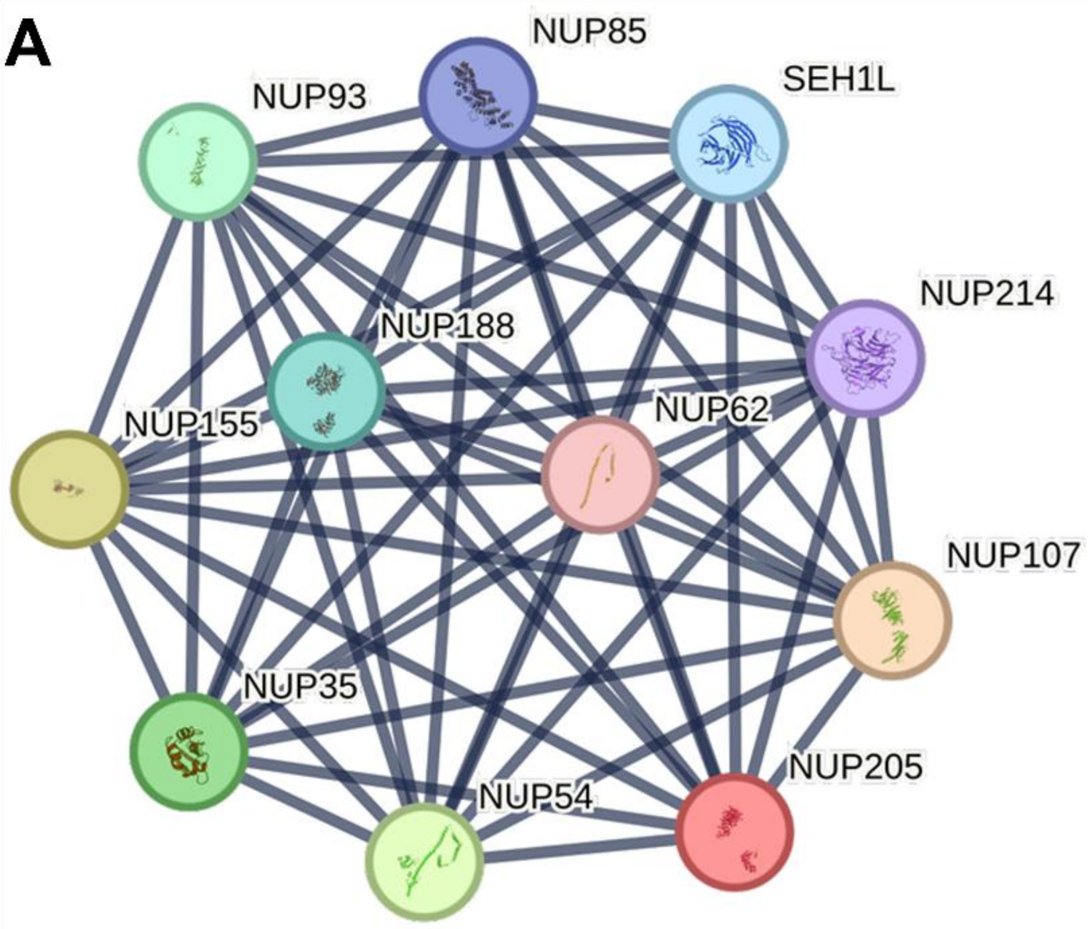
A
NUP85
SEH1L
NUP93
NUP214
NUP188
NUP62
NUP155
NUP107
NUP35
NUP205
NUP54

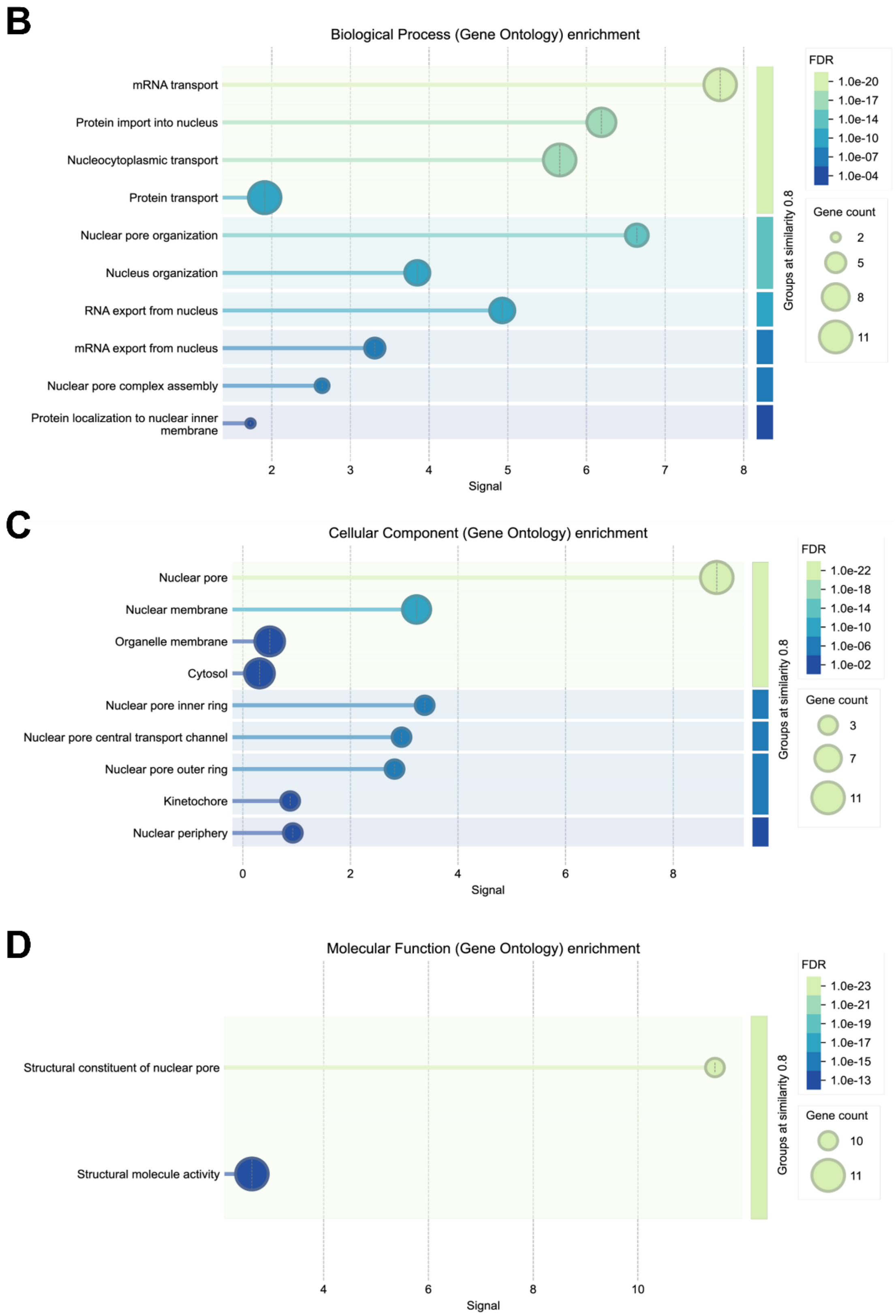

Figure S2. Protein–protein interaction and functional enrichment of NUP205 expression. (A) Protein–protein interaction of NUP205 expression. (B) Enriched GO-BP related to co-expressed proteins of NUP205. (C) Enriched GO-CC related to co-expressed proteins of NUP205. (D) Enriched GO-MF related to co-expressed proteins of NUP205.
